# Genes of the antioxidant system of the honey bee: annotation and phylogeny

**DOI:** 10.1111/j.1365-2583.2006.00695.x

**Published:** 2006-10-01

**Authors:** M Corona, G E Robinson

**Affiliations:** Department of Entomology, University of Illinois at Urbana-Champaign USA

**Keywords:** Antioxidant genes, honey bee genome

## Abstract

Antioxidant enzymes perform a variety of vital functions including the reduction of life-shortening oxidative damage. We used the honey bee genome sequence to identify the major components of the honey bee antioxidant system. A comparative analysis of honey bee with *Drosophila melanogaster* and *Anopheles gambiae* shows that although the basic components of the antioxidant system are conserved, there are important species differences in the number of paralogs. These include the duplication of thioredoxin reductase and the expansion of the thioredoxin family in fly; lack of expansion of the Theta, Delta and Omega GST classes in bee and no expansion of the Sigma class in dipteran species. The differential expansion of antioxidant gene families among honey bees and dipteran species might reflect the marked differences in life history and ecological niches between social and solitary species.

## Introduction

Reactive oxygen species (ROS) are constantly generated as by-products of aerobic metabolism. Accumulated evidence suggests that oxidative damage to cellular components induced by ROS is a major contributive cause of degenerative diseases and ageing. ROS generation occurs mainly in mitochondria in which more than 90% of the oxygen used by the cell is consumed (Perez-Campo *et al*., 1998). Aerobic organisms have evolved a complex network of enzymatic and non-enzymatic antioxidant systems to avoid oxidative damage. Key components of the antioxidant defence system are conserved throughout evolution, but there are unique adaptations among different groups. The major changes in insects in comparison with vertebrates and other phylogenetic groups include the loss of genes encoding functional glutathione reductase (GR) and glutathione peroxidase (GPX). Homologous genes for thioredoxin reductase (TrxR) (Kanzok *et al*., 2001) and thioredoxin peroxidase (TPX) (Radyuk *et al*., 2001) activities, respectively, act in their place.

There are both primary and secondary antioxidant enzymes, which act directly or indirectly on ROS molecules. The first line of defence against ROS attack is provided by three different kinds of primary antioxidant enzymes that act directly on ROS: (1) superoxide dismutases (SODs), which rearrange superoxide to oxygen and hydrogen peroxide; (2) catalase, which prevents free hydroxyl radical formation by breaking down hydrogen peroxide into oxygen and water; and (3) peroxidases, which catalyse an analogous reaction in which hydrogen peroxide is reduced to water by a reductant that acts as an electron donor, normally reduced thioredoxin (TRX) or glutathione (GSH). In addition, insects have three families of genes that encode antioxidant enzymes that act as peroxidases: TPXs, also known as peroxiredoxins (Radyuk *et al*., 2001), phospholipid-hydroperoxide GPX homologs with thioredoxin peroxidase activity (GTPX) (Missirlis *et al*., 2003), and glutathione S-transferases (GSTs) (Tang & Tu, 1994; Toba & Aigaki, 2000). Secondary antioxidant enzymes that act indirectly on ROS include TrxR, which recycles both TRX and GSH (Kanzok *et al*., 2001), and methionine sulphoxide reductases (MsrA and MsrB), which are involved in protein reparation by catalysing the TRX-dependent reduction of methionine sulphoxide to methionine (Moskovitz *et al*., 1996; Kumar *et al*., 2002).

Honey bee antioxidant enzymes are of particular interest because of their potential involvement in some of the exceptional biological characteristics of the queen honey bee, especially its longevity relative to worker bees (10 × longer; e.g. Page & Peng, 2001). Elevated expression of several traditional antioxidant-encoding genes occurs in young queens and old workers (Corona *et al*., 2005), suggesting that queen longevity is not related to higher expression of these particular genes, a result consistent with findings for Sod1 in *Lasius niger* ant queens (Parker *et al*., 2004). However, traditional antioxidants likely play roles in other processes. For example, Weirich *et al*. (2002) and Collins *et al*. (2004) reported that catalase, GST and SOD might contribute to the ability of queens to store sperm in their spermatheca for several years without loss of viability.

The recent release of the honey bee genome sequence provides the first opportunity to compare the whole set of antioxidant genes between insect orders. In this report we present the results of the manual annotation of the main antioxidant genes of *Apis mellifera*, a hymenopteran social insect, and a comparative analysis with the dipteran *Anopheles gambiae* and *Drosophila melanogaster*.

## Results and discussion

We identified 38 antioxidant genes in the honey bee genome, which include all major components of the enzymatic antioxidant system. This report does not include the annotation of genes encoding proteins thought to have indirect antioxidant effects mediated by metal binding capacities, such as vitellogenin (Seehuus *et al*., 2006), transferrin (Kucharski & Maleszka, 2003; do Nascimento *et al*., 2004), ferritin (Dunkov & Georgieva, 1999; Geiser *et al*., 2003) and metallothioneins (Egli *et al*., 2006).

In general, antioxidant genes encode small proteins less than 250 amino acids, with the exception of TrxR, catalase and proteins of unknown function such as Rsod and Trx/Grx-like proteins, which probably diverged by duplication of ancestral *Cu/ZnSOD* and *Trx*/glutaredoxin (*Grx*) genes. Most of the honey bee's antioxidant genes have protein-encoding regions with high A/T content (64% average, Table 1), a characteristic that is not specific to antioxidant genes, but rather is a general attribute of the honey bee genome. The honey bee genome is reported to contain 67% A/T, compared with 58% in *D. melanogaster* and 56% in *Anopheles gambiae* (Honey Bee Genome Consortium, 2006). It has been postulated that genes from organisms with high rates of metabolism use more A-ending codons than those from organisms with lower rates (Xia, 1996). This hypothesis has not yet been studied in insect species, which in general have very high metabolic rates (Suarez *et al*., 2000).

**Table 1:** Summary of honey bee antioxidant gene annotation. Gene localization based on the scaffolds_assembly_2 database. *Apis mellifera GstO2* and *Gstu1* are partial sequences

Gene	aa	Location	introns	ORF AT%
*Sod2*	218	Group11.11	2	65.3
*Sod1*	152	Group8.3	3	61.0
*Sod3*	178	GroupUn	2	64.2
*CCS*	266	GroupUn.5386	6	70.8
*Rsod*	1100	GroupUn.153	18	69.7
*Cat*	513	Group6.23	7	61.6
*Gtpx1*	168	GroupUn.5	3	68.4
*Gtpx2*	201	Group5.15	1	70.3
*Tpx1*	194	GroupUn.29	2	63.4
*Tpx3*	242	Group15.12	2	66.9
*Tpx4*	220	GroupUn.1374	4	61.8
*Tpx5*	220	Group12.14	3	67.0
*Tpx6*	219	Group9.2	1	57.2
*GstT1*	230	GroupUn.336	3	72.0
*GstD1*	217	Group15.2	4	56.1
*GstS1*	204	GroupUn.1306	3	66.0
*GstS2*	202	GroupUn.1306	2	63.4
*GstS3*	207	GroupUn.898	3	60.3
*GstS4*	206	Group4.16	3	58.6
*GstZ1*	217	Group5.15	4	63.3
*GstO1*	241	Group1.28	4	66.9
*GstO2*	partial	GroupUn.264	4	ND
*Gstu1*	partial	GroupUn.176	ND	ND
*Gstmic1*	149	Group2.5	1	70.4
*Gstmic2*	156	Group1.56	1	53.6
*Trxr-1*	494	GroupUn.68	7	64.6
*Trx-1*	105	GroupUn.35	3	68.3
*Trx-2*	136	Group6.16	2	64.9
*Trx-3*	103	GroupUn.125	0	73.5
*Trx-like1*	287	Group14.6	3	66.1
*Trx-like2*	488	Group3.21	5	48.0
*Trx-like3*	411	Group13.2	5	65.6
*Grx1*	98	GroupUn.505	1	67.0
*Grx2*	133	Group11.6	2	65.2
*Grx-like1*	711	Group6.26	0	43.2
*Trx/Gtx*	222	Group15.14	1	67.9
*MsrA*	217	GroupUn.104	3	65.7
*MsrB*	137	GroupUn.304	2	59.6

ND, not determined.

### Comparative analysis of A. mellifera, D. melanogaster and A. gambiae antioxidant genes

#### Superoxide dismutases

SOD converts radical superoxide to oxygen and hydrogen peroxide, providing the first line of defence against ROS produced in the mitochondria. SODs normally exist in two forms in eukaryotic cells; the two forms differ in cellular localization and in the structure of their active sites. MnSOD (SOD2) is present in the inner mitochondrial space and Cu/ZnSOD (SOD1) in the cytoplasm. Like most eukaryotes, honey bees have a single mitochondrial MnSOD gene located on chromosome 11. Vertebrate orthologs, including those in Tetraodon and human, have higher overall identity with the honey bee ortholog (66.21 and 62.33% ID) than dipteran species (*Drosophila*, 59.09, *Anopheles* 59.17). Possible explanations for this phylogenetic discordance include rapid divergence of the dipteran orthologs (Honey Bee Genome Sequencing Consortium, 2006).

The Cu/ZnSOD family includes five members in *Drosophila* and *Anopheles* and four members in *Apis* (Table 2). In *Drosophila* this group includes the canonical cytoplasmatic Cu/ZnSOD (CG11793), extracellular SOD (*Sod3*, CG9027), copper chaperone (CCS, CG17753), related to Sod (*Rsod*, CG31028), and Sodesque (*Sodq*, CG5948). Extracellular CuZnSODs are present in several animal groups, from nematode to mammals. In insects, they have been identified in *D. melanogaster*, *Anopheles gambiae* (Landis and Tower, 2005) and *Lasius niger* (Parker *et al*., 2004). The honey bee has an extracellular Cu/ZnSOD (SOD3) of 178 amino acids.

**Table 2:** Major components of the enzymatic antioxidant system of *Apis mellifera*, *Drosophila melanogaster* and *Anopheles gambiae*. Gene identification numbers: for bee, the BeeBase ID; for mosquito, the Genbank accession number; for fly, the Flybase gene ID. NP indicates genes with no automatic prediction in bees. For the *GST Delta* and *Epsilon* classes of *Drosophila* and *Anopheles*, only four representative members are shown

Gene	Apis	Anopheles	Drosophila
*Sod2*	GB14346	AAS17758	CG8905
*Sod1*	GB10133	AAR90328	CG11793
*Sod3*	NP	AAS17758	CG9027
*CCS*	GB14210	XP_308747	CG17753
*Rsod*	GB14567	EAA00894	CG31028
*Sodq*	Not identified	EAA04552	CG5948
*Cat*	GB11518	XP_314995	CG6871
*Gtpx1*	GB14138	XP_313166	CG12013
*Gtpx2*	GB18955	XP_562772	Not identified
*Gpx-like*	Not identified	Not identified	CG15116
*Tpx1*	GB19380	XP_308081	CG1633
*Tpx2*	Not identified	XP_308336	CG1274
*Tpx3*	GB10972	XP_565975	CG5826
*Tpx4*	GB10498	XP_320690	CG12405
*Tpx5*	GB10803	XP_308753	CG3083
*Tpx6*	NP	Not identified	CG6888
*GstT1*	GB12047	AAM61893, AAM61892	CG1702, CG30005
			CG30000, CG1681
*GstD1*	GB18045	AAC79995	CG10045
*GstD2-12*	Not identified	CAA96104, AAM53610	CG4181, CG4381
		AAM53607, AAM53607	CG11512, CG12242
*GstS1*	GB16959	AAA29358	CG8938
*GstS2*	NP	Not identified	Not identified
*GstS3*	GB19254	Not identified	Not identified
*GstS4*	GB14372	Not identified	Not identified
*GstZ1*	GB17672	AF515522	CG9363
*GstZ2*	Not identified	Not identified	CG9362
*GstO1*	GB11466	AAP13482	CG6781
*GstO2*	GB19678		CG6662
*GstO3-4*	Not identified	Not identified	CG6776 CG6673
*GSTu1*	GB15512	AAM61888	CG33546
*GstE1-13*	Not identified	AAG45163, AAG45164	CG5164 CG17524
		AAL59653, AAL59654	CG17523 CG17525
*GSTmic1*	GB12371	AAP37003	CG1742
*GSTmic2*	GB10566	AAP37005	CG33178
*Trxr-1*	GB14972	CAD30858	CG2151
*Trxr-2*	Not identified	Not identified	CG11401
*Trx-1*	GB17503	EAA04498	CG8993, CG8517
*Trx-2*	GB15855	EAA14495	CG31884, CG3315
			CG4193, CG13473
*Trx-3*	GB19972	EAA09650	CG3719
*Trx1-like1*	GB15457	EAA11972	CG5495
*Trx1-like2*	GB15572	XP_320264	CG14221
*Trx1-like3*	GB19276	XP_316887	CG9911
*Grx1*	GB10598	XP_309539	CG6852, CG7975
*Grx2*	GB18700	XP_312440	CG14407
*Grx-like1*	GB11664	EAA06446	CG31559, CG12206
*Trx/Gtx*	GB12870	EAA07378	CG6523
*MsrA*	GB10196	XP_320164	CG7266
*MsrB*	GB15486	XP_311902	CG6584

**Figure 1:**
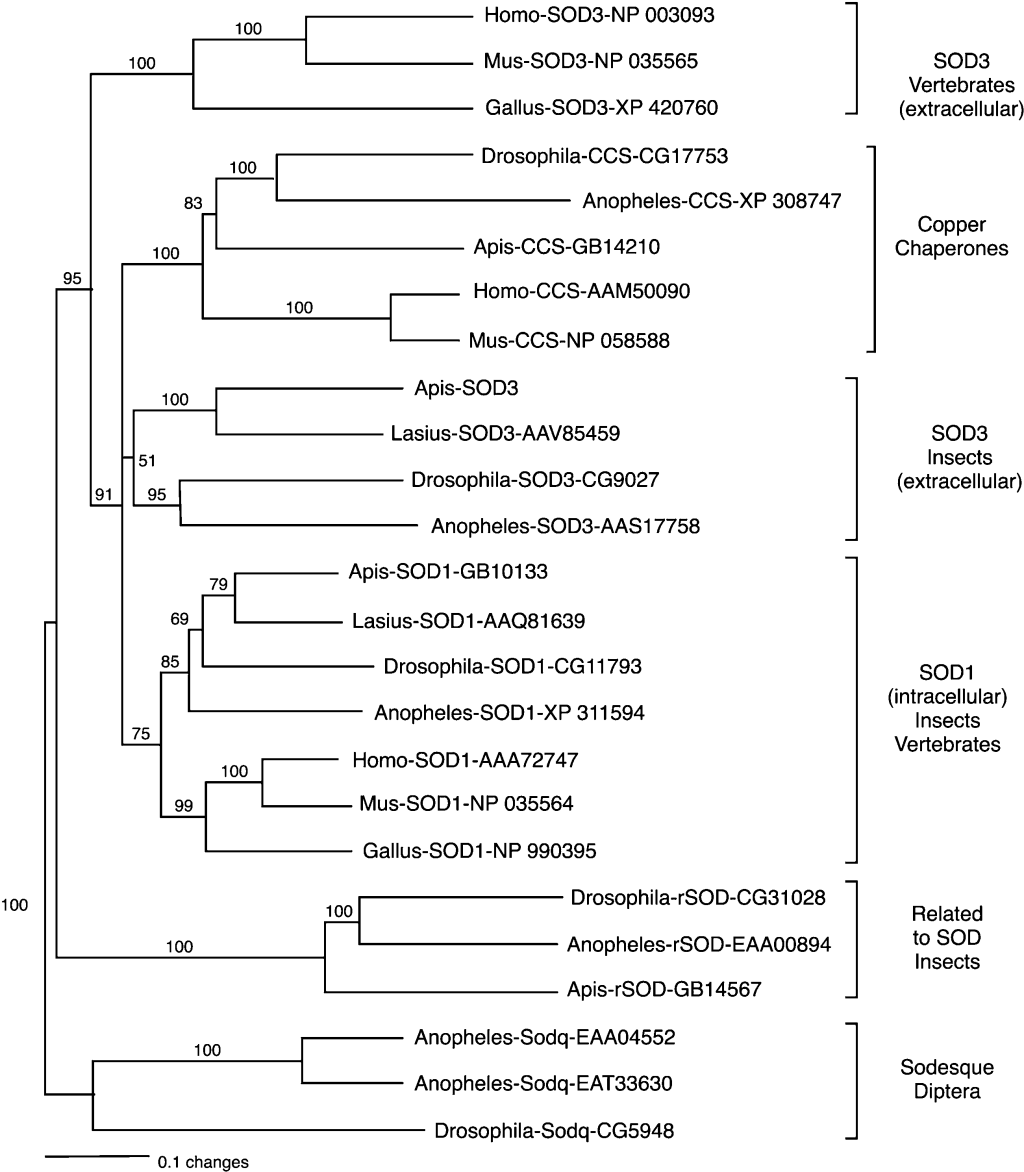
Neighbour joining tree showing the relationships of the CuZn SOD family. The GenBank accession number (*Anopheles gambiae*), Flybase ID (*Drosophila melanogaster*) and BeeBase ID (*Apis mellifera*) are shown for each sequence. Values above the branches represent bootstrap support.

Phylogenetic analysis (Fig. 1) shows that the extracellular SODs of insects and vertebrates form different monophyletic clades. This suggests the possibility that they evolved independently in each group, for example, by the addition of a signal peptide to cytoplasmatic SOD (Landis and Tower, 2005). Copper chaperone (CCS) has, in addition to the SOD domain, a N-terminal heavy-metal-associated domain (HMA) involved in the transport of copper to Cu/ZnSOD. As insect and vertebrate homologs form a single monophyletic clade, CCS proteins seem to have diverged from cytoplasmatic SOD before the separation of these two lineages.

A putative ortholog for the *Drosophila* Sodesque (*Sodq*) gene is present in *Anopheles gambiae*; however, it encodes a rapid evolving protein, with only 42% identity between these dipteran species. As a Sodq-related protein is also present in *Aedes aegypti* (EAT33630), but orthologs for this gene are absent in honey bee, other insects, and vertebrates, it is possible that this gene has diverged from cytoplasmatic SOD only in dipteran species. Sodq function in *Drosophila* is uncertain, because the fly ortholog lacks several conserved residues essential for catalytic function while possessing a signal peptide for extracellular targeting (Landis and Tower, 2005).

The *Drosophila* related to Sod gene (*Rsod*) is an atypical member in the Cu/ZnSOD family. It has a duplicated SOD domain and an unusually high number (18) of introns (Table 1). Homologous genes (with two or three SOD domains) are present in *Anopheles*, *Apis*, protozoa (*Dictyostelium discoideum* XP_639320 and XP_639300), fish (*Tetraodon nigroviridis*, CAF89944), but not in mammals. Rsod function is unknown in insects. However, a homologous protein (pernin, AAK20952) in *Perna canaliculus* (Mollusca) does not show SOD activity but might be involved in the transport of divalent metal cations (Scotti *et al*., 2001).

#### Catalase

Catalase prevents free hydroxyl radical formation by breaking down hydrogen peroxide into oxygen and water. A single catalase gene is normally present in eukaryotes, with the exception of *C. elegans*, in which this gene is duplicated. Honey bee catalase encodes a protein of 513 amino acids and is localized on chromosome 6. Catalase in *Apis*, as in other eukaryotes, is located in the cytosol and lacks a signal peptide necessary for secretion. Interestingly, catalase activity has been reported to be present in honey (White, 1975), which perhaps acts to keep H_2_O_2_ levels in honey (produced by bees as a preservative) below toxic levels. Since in the honey bee genome the only catalase is not extracellular, the source of the catalase in honey remains to be determined. It has been assumed that it comes from plants (White, 1975), but extracellular catalases are apparently only found in some bacteria and fungi. An intriguing possibility is that catalase in honey originates from endosymbiotic bacteria.

#### Thioredoxin peroxidases

**Figure 3:**
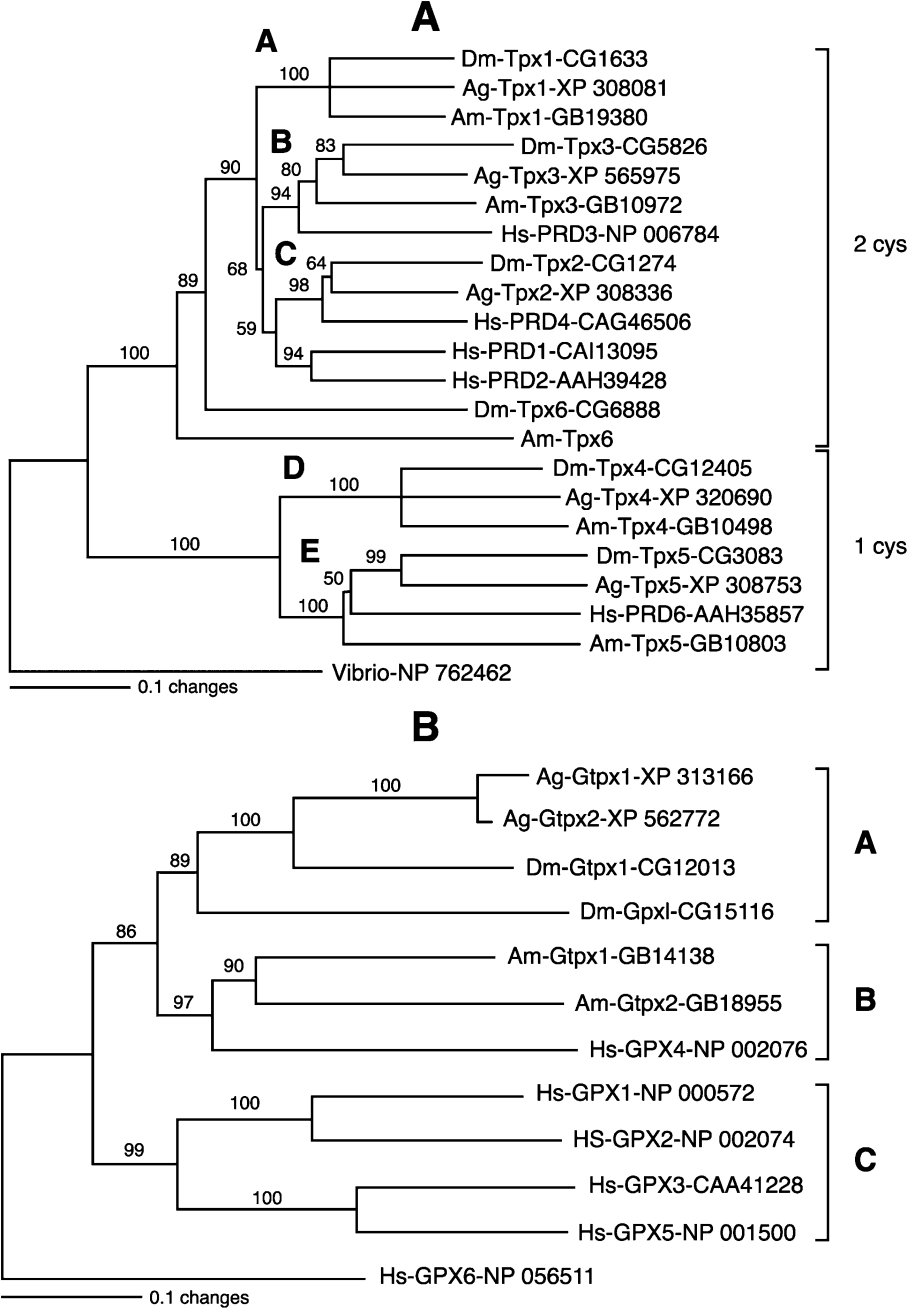
Neighbour joining tree showing the phylogenetic relationships of *Apis mellifera* (Am), *Anopheles gambiae* (Ag), *Drosophila melanogaster* (Dm) and *Homo sapiens* (Hs) peroxidases homologs. (A) Thioredoxin family. (B) Glutathione peroxidase homologs. Values above the branches represent bootstrap support.

TPXs, also known as peroxiredoxins, are a type of peroxidase that reduces H_2_O_2_ using electrons provided by TRX (Chae *et al*., 1994). Based on the number of conserved cysteins, TPXs are classified into two subfamilies: 1-Cys and 2-Cys. In contrast to the 1-Cys, the 2-Cys subfamily has a second conserved Cys in the C-terminus (Trivelli *et al*., 2003) (Fig. 3A). The TPX family has five members in humans, which include cytosolic, mitochondrial and extracellular forms (Chang *et al*., 2004). The *Drosophila* genome also contains five TPX homologs (Radyuk *et al*., 2001) that comprise three cytosolic variants (*Tpx1*, CG1633, *Tpx4*, CG12405, *Tpx5*, CG3083), one mitochondrial (*Tpx3*, CG5826) and one secretable (*Tpx2*, CG1274).

**Figure 2:**
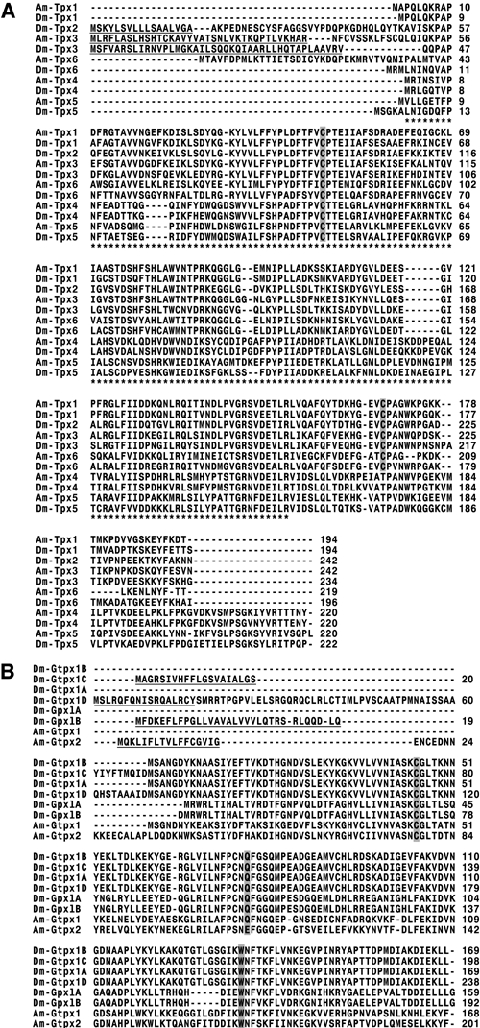
*Apis mellifera* and *Drosophila melanogaster* thioredoxin-dependent peroxidase homologs. (A) Thioredoxin peroxidase family (peroxiredoxins). Predicted signal peptide for Dm Tpx-2 (Dpx4156) and mitochondrial targeting peptide of *AmTpx3* and *DmTpx-3* (Dpx5037) are underlined. Asterisks mark the peroxiredoxin domain. Conserved cysteins are highlighted. (B) Glutathione peroxidase homologs with thioredoxin peroxidase activity. Predicted signal peptides (*AmGtpx2*, *DmGtpx1C*) and mitochondrial targeting peptides (*DmGtpx1D*) are shown underlined. Amino acids of the catalytic site (Ursini *et al*., 1995) are highlighted. Amino acid colour follows the ClustalW code.

We identified a new putative TPX homolog in *Drosophila* (*DmTpx6*, CG6888), five Tpx members in *Anopheles* and five homologs in *Apis* (Table 2). Compared with dipteran species, honey bee seems to have lost the secretable variant (*Tpx-2*). *AmTpx6* and *DmTpx6* are the more diverged members of the Cys-1 subfamily; there is no mosquito homolog (Fig. 2A and 3A). Phylogenetic analysis (Fig. 3A), showed that the different insect and human homologs are grouped in separate phylogenetic groups. Three of them are included in the 2-Cys subfamily and two in the 1-Cyst subfamily. This distribution suggests that the major members of the TPX family could have diverged before the separation of the insect and vertebrate metazoan ancestor. Consistent with this analysis is the finding that each of the phylogenetic groups contain members that seem to have conserved their particular subcellular localization. Clades A, D and E contain cytoplasmic, clade B contains mitochondrial, and clade C contains extracellular variants (as inferred in *Apis mellifera* and *Anopheles gambiae* by the presence of predicted mitochondrial targeting and signal peptides).

#### Glutathione peroxidase homologs

GPX catalyses the reduction of hydrogen peroxide and organic hydroperoxides. In mammals, GPX catalyses the reduction of hydroxyperoxides utilizing GSH as an electron donor (Ursini *et al*., 1995). Early work (Smith & Shrift, 1979) found that insects lack GPX activity. However, the *Drosophila* genome contains two GPX homologs. One of these genes encodes for an enzyme that uses TRX, rather than GSH, as an electron donor and was therefore referred to as a GPX homolog with TPX activity, *Gtpx-1* (CG12013) (Missirlis *et al*., 2003). This gene also is known as *DmPHGpx* and has been shown to be highly expressed in testis (Li *et al*., 2003). The second *Drosophila* GPX homolog remains to be biochemically characterized and is referred to as *GPX-like* gene (*Gpxl*, CG15116).

We found that both *Apis mellifera* and *Anopheles gambiae* also have a pair of GPX homologs (Table 2), although one of the honey bee homologs (*AmGtpx-2*, GB18955) lacks one of the three conserved residues of the catalytic site (Fig. 2B) (Ursini *et al*., 1995). Homologs in each species share more identity with each other than with homologs in other species, suggesting that they are paralogs that diverged after speciation. As might be expected, the dipteran homologs are more closely related to each other (Fig. 3B, clade A) compared with those of the honey bee, which form a monophyletic group (clade B) with human *Gpx4*. Our phylogenetic analysis also shows that each pair of homologous genes in mosquito and bee are more closely related to each other compared with the pair of GPX homologs in *Drosophila*. This could be due to several causes, including the possibilities that the duplication event occurred early in *Drosophila* or there was rapid sequence divergence of the Gpxl gene.

Humans have six GPX homologs, some with cytosolic, mitochondrial or extracellular localization. In *Drosophila* there are four *Gpx-1* isoforms, two of them with putative cytosolic (CG12013-PA, CG12013-PB), one with mitochondrial (CG12013-PD) and one with extracellular localization (CG12013-PC), as inferred by computational identification of putative mitochondrial targeting sequences and signal peptides. This suggests that diversity in subcellular localization in *Drosophila* is achieved via alternative splicing rather than gene duplication, and honey bee may share a similar gene expression strategy (Table 3).

**Table 3:** Predicted subcellular localization and available expression data for honey bee antioxidant genes. Putative mitochondrial and extracellular variants were inferred by computational identification of predicted mitochondrial targeting and secretory signal peptides

Gene	Localization	W	Q
*Sod2*	M	BTA	BTA
*Sod1*	C	BTA	BTA
*Sod3*	E	B	
*CCS*	C		
*Rsod*	E		
*Cat*	C	BTA	BTA
*Gtpx1*	C	BTA	BTA
*Gtpx2*	E		
*Tpx1*	C	B	
*Tpx3*	M	BTA	BTA
*Tpx4*	C	B	
*Tpx5*	C		
*Tpx6*	C		
*GstT1*	C		
*GstD1*	C	BTA	BTA
*GstD2-12*	C		
*GstS1*	C	B	
*GstS2*	C	B	
*GstS3*	C	B	
*GstS4*	C		
*GstZ1*	C		
*GstZ2*	C		
*GstO1*	M	B	
*GstO2*	Unk		
*GstO3-4*	C		
*Gstu1*	C		
*GstE1-13*	C		
*Gstmic1*	Mic		
*Gstmic2*	Mic		
*Trxr-1*	C	BTA	BTA
*Trx-1*	M		
*Trx-2*	C		
*Trx-3*	C		
*Trx1-like1*	C		
*Trx1-like2*	C		
*Trx1-like3*	C	B	
*Grx1*	C		
*Grx2*	M		
*Grx-like1*	N	B	
*Trx/Gtx*	C	B	
*MsrA*	C	BTA	BTA
*MsrB*	C		

Cellular localization: C, cytosolic; M, mitochondria; E, extracellular. N, nuclear; Mic, microsomal; Unk, unknown (5′ truncated genes). Honey bee castes: W, workers; Q, queens. Tissues: B, brain; T, thorax; A, abdomen.

Like *Gpx-1*, the second *Drosophila* GPX homolog (*Gpxl*) also has a splicing variant with a putative signal peptide sequence (CG15116-PB), and a splice variant with a putative signal peptide sequence occurs for at least one of the *Apis* (*AmGtpx1*, GB18955) and *Anopheles* (*Ag Gtpx-1*, XP_313166) *Gpx-like* genes (Table 3). Thus, it is likely that at least one of the two paralogs in each species have an extracellular function, as it is the case for four of six human *Gpx* genes (Lee *et al*., 2005). At present the function of the putative extracellular GPX-like proteins in insects is unknown. Interestingly, an extracellular GPX homolog with no enzymatic activity was found in the parasitic wasp *Venturia canescens* that is included in a virus-like particle injected with the eggs into the host, and is probably involved in protection of the egg (Li *et al*., 2003).

#### Thioredoxin reductase

TrxR is an essential enzyme that in insects transfers reducing equivalents from NADPH to thioredoxin (TrxS_2_) and GSH disulphide (GSSG). The resulting products, Trx (SH)_2_ and GSH, respectively, act as thiol-based reductants and powerful intracellular antioxidants (Holmgren, 1989). Mammal TrxR carries a distinctive COOH-Terminal extension that includes a tetrapeptide motif (Gly-Cys-Sec-Gly-OH) containing a selenium in the form of selenocysteine (s residue) involved in TRX reduction. This motif distinguishes TrxR proteins from other structural and functionally closely related flavoprotein disulphide oxidoreductases such as lipoaminede hydrogenases and ferredoxin reductases (Nordberg & Arner, 2001). The *Drosophila* ortholog (*Trxr-1*) has a cysteine instead of selenocysteine, with equivalent function (Kanzok *et al*., 2001). As *Anopheles* orthologs also have a cysteine residue in this site (Bauer *et al*., 2003) the absence of selenium-containing TrxR might be general characteristic of dipteran species.

**Figure 4:**
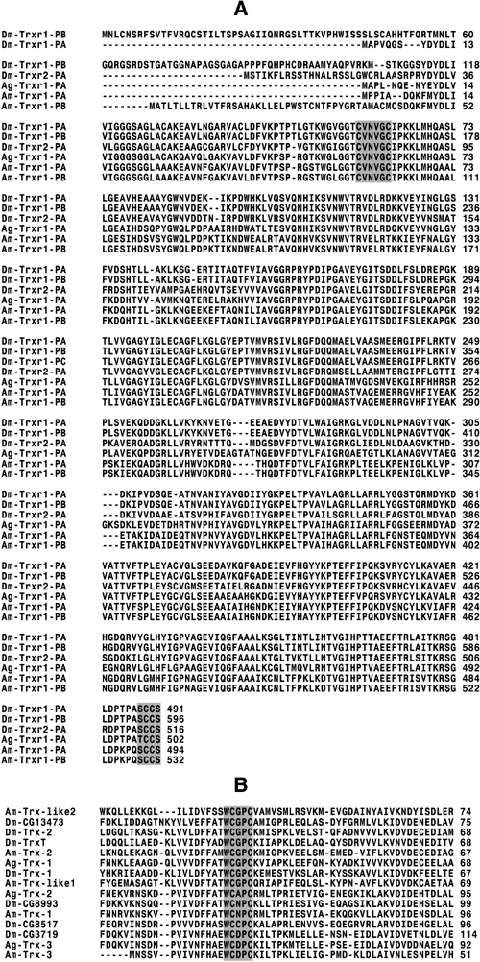
Alignments for thioredoxin reductases and thioredoxins from *Apis mellifera* (Am), *Drosophila melanogaster* (Dm) and *Anopheles gambiae* (Ag). (A) Thioredoxin reductase family. The sequences of redox-active centres are highlighted. (B) Fragment of an alignment of thioredoxin family proteins. The conserved active site (CXXC) (Holmgren, 1989) is highlighted.

In contrast with human, which has three *Trxr* genes, and *Drosophila*, which has two, *Apis* and *Anopheles* have only a single *Trxr* gene (Table 2, Fig. 4A). In *Drosophila*, *Trxr-1* encodes three splice variants that include one mitochondrial and two cytoplasmic forms (Missirlis *et al*., 2002). The functional significance of the second *Drosophila* *Trxr* gene (*Trxr2*) is unknown, but it encodes a protein with a potential mitochondrial targeting peptide. *Anopheles* has a single *Trxr* gene, and, as in the *Drosophila* ortholog has three splice variants encoding for one mitochondrial and two cytoplasmic forms (Bauer *et al*., 2003). *Apis* also has a single *Trxr* gene (Table 2); we identified two putative splice variants, but none of them appear to encode a mitochondrial variant. We were unable to localize an alternative 5′ exon encoding a mitochondrial targeting peptide. However, as a mitochondrial TrxR is necessary to provide reduced TRX for mitochondrial peroxidases (including at least Tpx3) and given that catalase is not expressed in mitochondria to reduce H_2_O_2_, a mitochondrial variant should be present.

#### Thioredoxins

TRXs are small, highly conserved oxidoreductase proteins required to maintain the redox homeostasis of the cell. TRX is reduced by TrxR through NADPH (Holmgren *et al*., 2005). In mammals seven TRX/TRX-like proteins have been identified, including tissue-specific and ubiquitously expressed forms with cytoplasmic, mitochondrial and Golgi apparatus-associated variants (Spyrou *et al*., 1997; Miranda-Vizuete *et al*., 2001; Jimenez *et al*.,^ 2004, 2006^). In *Drosophila* three *Trx* genes have been characterized: Trx-1 (*deadhead* gene, CG4193) (Pellicena-Palle *et al*., 1997; Kanzok *et al*., 2001), *Trx-2* (CG31884) (Bauer *et al*., 2002) and *TrxT* (CG3315) (Svensson *et al*., 2003). Whereas *Trx-1* and *TrxT* are localized in the nucleus and are ovary- and testis-specific, respectively, *Trx-2* is localized in the cytoplasm of somatic tissues. This distribution suggests that *Trx-2* plays a major part in whole-body redox homeostasis. Accordingly, *Trx-2* but not *Trx-1*, functions as a substrate for TrxR (Bauer *et al*., 2002).

The *Drosophila* genome contains four additional genes (CG8993, CG8517, CG3719, CG13473) that contain both an overall TRX-like fold domain (Martin, 1995) and the conserved motif Cys-X_1_X_2_-Cys of the active site (Holmgren *et al*., 2005). Two of these genes (CG8993 and CG8517) encode for proteins with probable mitochondrial targeting peptides. The *Anopheles* genome contains at least three putative *Trx* genes, one with cytoplasmic localization (*Txr-1*, EAA14495) (Bauer *et al*., 2002) and two with probable mitochondrial localization (*Trx-2*, EAA04498 and *Trx-3*, XP_314234).

**Figure 5:**
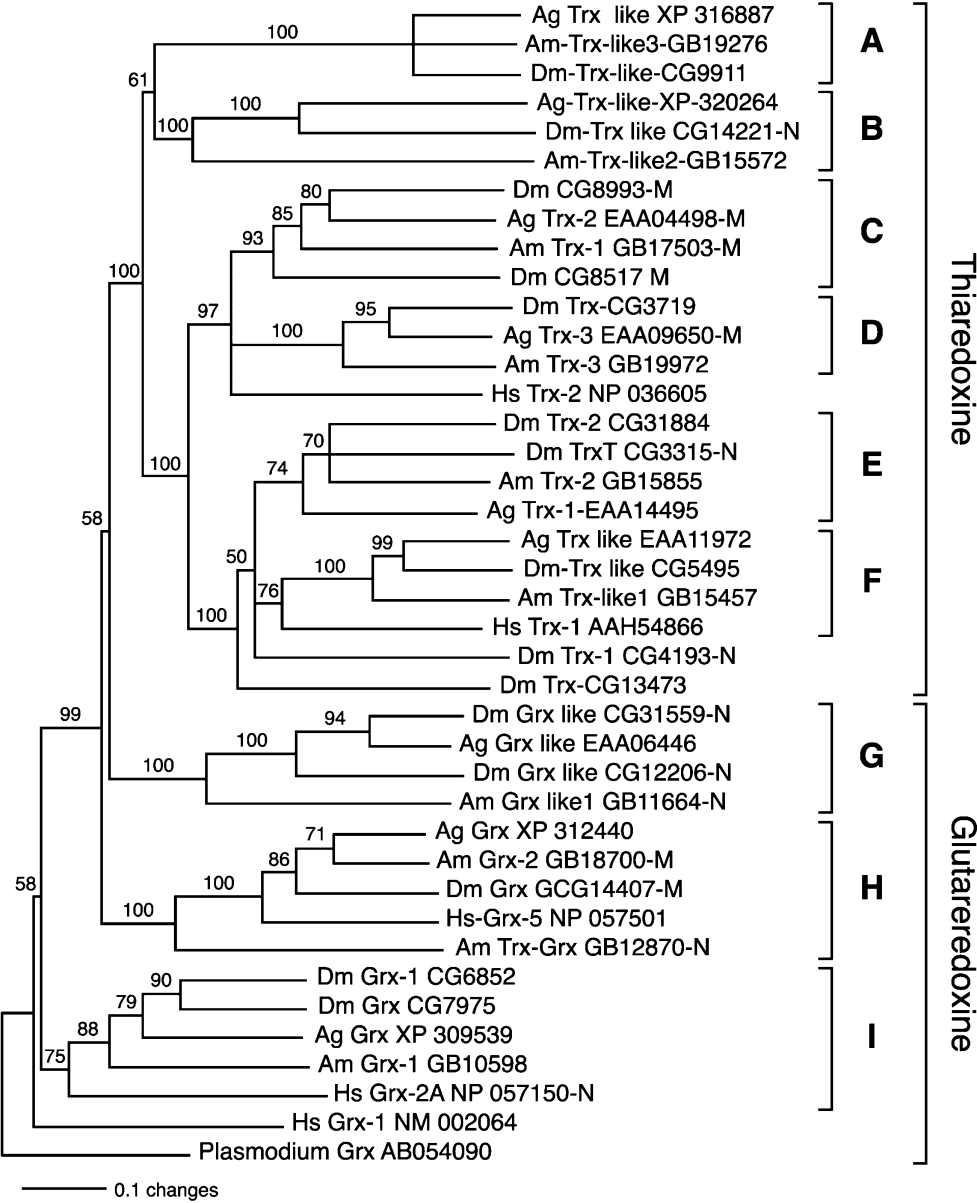
Phylogenetic tree of the thioredoxin/glutharedoxin protein family. ‘M’ and ‘N’ after the accession number indicate mitochondrial or nuclear predicted subcellular localization. Values above the branches represent bootstrap support.

As in *Anopheles*, the *Apis* genome contains three genes encoding putative TRX homologs: Am Trx-1 (GB17503) with predicted mitochondrial localization and an apparent ortholog of *Drosophila* CG8993 and *Anopheles* *Trx-2* (clade C, Fig. 5); *AmTrx-2* (GB15855), a putative ortholog of *Drosophila* *Trx-2* (60.38% ID) and *Anopheles* Trx-1 (56.6% ID) (clade E) and the intronless gene Am Trx3 (GB19972), putative ortholog of *Drosophila* CG3719 and *Anopheles* Trx3, suggesting that it is not of bacterial origin (clade D). Thus, each TRX homolog in honey bee and mosquito has a corresponding putative ortholog in fly. But *Drosophila melanogaster* has four additional genes with no apparent ortholog in honey bee and mosquito. These genes include CG8517, which seems to have duplicated from CG8993, *Trx-1*, *TrxT* and CG13473, which possibly diverged from *Drosophila* Trx-2 after fly and mosquito diverged from the common dipteran ancestor. Thus, compared with *Apis* and *Anopheles*, the TRX subfamily in *Drosophila* was clearly expanded.

As in other organisms, insect genomes also contain a large group of genes encoding TRX-related proteins containing one or multiple TRX domains, which include protein disulphure isomerases (Arner & Holmgren, 2000) and other proteins of unidentified function. One group of these proteins, which have higher identity to *bona fide* TRX, contain a single N-terminal TRX domain, but have an additional C-terminal extension of unknown function. One homolog of this protein in humans, TRX-like-1 (TXL-1), is a substrate for the cytosolic selenoprotein TrxR-1 (Jimenez *et al*., 2006). We identified three genes encoding this kind of TRX-like protein with homologs in *Apis*, *Anopheles* and *Drosophila* genomes (Table 2, Fig. 5 clades A, B and F). Only two of them (*Trx-like-1* and *Trx-like 2*) have a TRX domain with a conserved CXXC active site (Fig. 4B).

#### Glutaredoxin

GRXs are both structurally and functionally related to TRXs. Insect genomes contain genes encoding GRX homologs, although at present their products have not been characterized. In most organisms oxidized GRX proteins are regenerated by reduced GSH, and the resulting oxidized GSH (GSSG) is reduced by GSH reductase (Holmgren *et al*., 2005). However, in insects the reduction of GSSG is performed by TrxR (Kanzok *et al*., 2001). In vertebrates, the products of three *Grx* genes have been characterized: GRX1, GRX2 (Johansson *et al*., 2004) and the more distantly related, GRX5 (Wingert *et al*., 2005). In humans, GRX1 is localized primarily in the cytoplasm, whereas *Grx2* encodes for both nuclear and mitochondrial variants (Johansson *et al*., 2004; Holmgren *et al*., 2005). In zebrafish GRX5 is primarily localized in mitochondria (Wingert *et al*., 2005), although in human the reported uncharacterized homolog (NP_057501) lacks a potential mitochondrial targeting peptide.

In *Apis*, we identified two GRX homologs that we named *Grx1* (GB10598) and *Grx2* (GB18700), with predicted cytoplasmic and mitochondrial localizations, respectively. *Grx1* forms a monophyletic group (Clade I, Fig. 5) with one human (*Grx2*, NP_057150), one *Anopheles* (XP_309539) and two *Drosophila* (CG6852, CG7975) homologs. This suggests that *Grx1* was duplicated only in flies. *Grx2* has putative orthologs in human (*Grx5*, NM_016417), *Drosophila* (GCG14407) and *Anopheles* (XP_312440). Although this group of proteins shares a clear common evolutionary origin with other GRX proteins, members of this group contain a single cysteine residue at the putative active site (Rodriguez-Manzaneque *et al*., 1999).

Insect genomes contain two additional groups of genes encoding GRX-related proteins of unknown function (*Grx-like* genes). The first group contains a GRX domain in the C-terminal of the predicted protein and has a predicted nuclear localization. In honey bees this group is represented by *Grx-like-1*, which forms a monophyletic group with two *Drosophila* and one *Anopheles* homologs (Clade G, Fig. 5). The other group of *Grx-like* genes, with orthologs in honey bee (GB12870), fly (CG6523) and mosquito (EAA07378), is interesting because it encodes proteins that contain a TRX domain in the N-terminal region and a GRX domain in C-terminal region.

#### Glutathione S-transferases

GSTs are multifunctional proteins essential for xenobiotic metabolism and protection against peroxidative damage. The GST superfamily can be divided into several structurally and functionally classes that show unique variations among different phylogenetic groups. Plants have exclusive Tau and Phi classes, whereas mammalian have the mitochondrial Kappa class. In insects eight different classes have been identified: Epsilon (GSTe), Delta (GSTd), Theta (GSTt), Zeta (GSTz), Omega (Gsto) and Sigma (GSTs), the structurally unrelated microsomal class (GSTmic) and the denominated unclassified class (u), so designated for the lack or precise immunological or biochemical data (Ding *et al*. 2003). Most studies of GSTs in insects have been focused on their role in conferring insecticide resistance. (Claudinos *et al*. in press) have recently analysed the GST family in honey bees from this perspective. GST can be considered a primary antioxidant enzyme, given the fact that at least the Delta (Tang & Tu, 1994), microsomal (Toba & Aigaki, 2000), and Sigma classes (Singh *et al*., 2001) exhibit GPX activity with cumene hydroperoxide.

**Figure 6:**
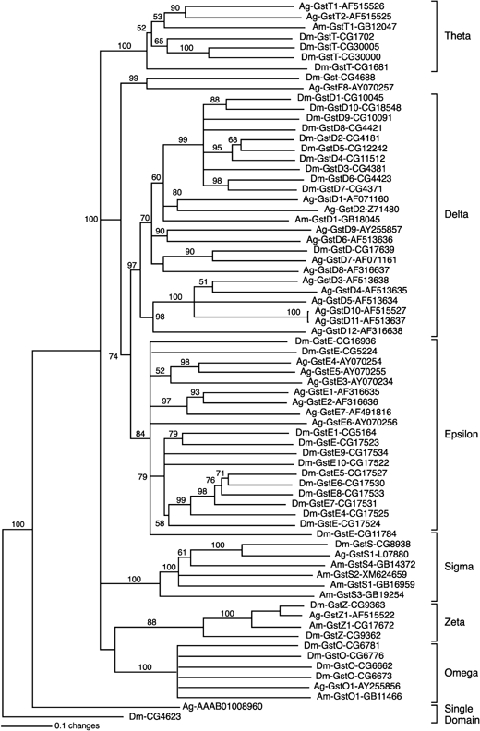
Phylogenetic relationships of GST family. GSTs belonging to the unclassified (Ding *et al*., 2003) class were not included. Values above the branches represent bootstrap support. Each entry has a species name (Am, for *A. mellifera*; Ag, for *A. gambiae*; Dm, for *D. melanogaster*), GST class, number if assigned, and accession number.

The GST superfamily includes 43 members in *Drosophila* and 37 in *Anopheles*. (Ding *et al*., 2003). In contrast, we only identified 12 genes in the *Apis* genome (two of them with partial sequences, Table 1) Compared with dipteran species, which experienced considerable expansion of the Delta and Epsilon GTS subfamilies, the bee genome contains a single ortholog of the Delta class and no members of the Epsilon class. Another difference includes double and single duplications in the Omega and Zeta classes that occurred only in fly. In addition, the Theta class ortholog that experienced two duplications in fly and one in mosquito was apparently not duplicated in bee (Table 1 and Fig. 6).

**Figure 7:**
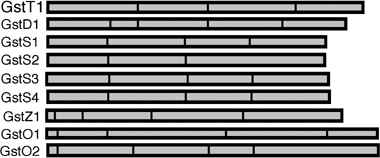
Intron position in *Apis mellifera* GST family members. With the exception of the third intron of *GstS2*, intron positions are conserved between the members of the Gst Sigma class. *GstO2* genomic sequences is truncated toward the deduced C terminal region.

The Sigma class is the only GST lineage larger in honey bees in comparison with dipteran species. There are four members of this group in bee and a single ortholog in fly and mosquito. This is also the group with the higher conservation in intron position (Fig. 7). In addition, two members of this group (*GstS1–2*) are the only antioxidant genes so far found to be physically located close to each other (Table 1). Both findings suggest that in bees the GST Sigma class could have been expanded by a recent duplication event, as seems to be the case for the Delta and Epsilon classes in *Drosophila* (Sawicki *et al*., 2003) and *Anopheles* (Ding *et al*., 2003). Lack of knowledge of endogenous insect GST substrates makes it difficult to interpret the functional consequences deriving from the differential expansion of GST subfamilies between dipteran species and honey bees. Perhaps they reflect both differences in metabolic activity and variation in the quantity of pro-oxidant molecules ingested with the food. For example, the Epsilon class (expanded in dipteran but lost in bees) is involved with DDT resistance (Ranson *et al*., 2000; Lumjuan *et al*., 2005) and is expected to be related to the detoxification of xenobiotics in general. It is reasonable to expect a higher quantity of xenobiotics in the food of a solitary species, with no parental care or sociality, compared with the food received by honey bees, especially during the larval stages and the first 2 weeks of adulthood, when their food is restricted to honey, pollen and glandular secretions provided by other members of the colony (Winston, 1987). In addition, honey bees feed on angiosperms in a highly mutualistic relationship; angiosperms have evolved many traits to attract bees for pollination purposes. Bees are much less likely to be exposed to naturally occurring feeding deterrents or toxins.

The expansion of the Sigma class, which occurred only in bees, seems to be involved with protection against oxidants produced by aerobic metabolism, rather than xenobiotics. In flies, these proteins are primarily located in the indirect flight muscles (Franciosa & Berge, 1995) and have been reported to play an important part in the detoxification of lipid peroxidation products (Singh *et al*., 2001). Honey bees take foraging trips that may last up to 1 h and they carry heavy loads of nectar and pollen during this time (Winston, 1987), so they likely produce a high level of free radicals (Young & Robinson, 1983). Perhaps this aspect of their life-style exerted selection on these detoxification genes.

#### Methionine-R-sulphoxide reductases

Methionine-R-sulphoxide reductases (Msr) are secondary antioxidant enzymes involved in protein repair, catalysing the TRX-dependent reduction of methionine sulphoxide to methionine (Moskovitz *et al*., 1996). Methionine sulphoxides can be reduced to methionines by methionine-S-sulphoxide reductase (MsrA) and methionine-R-sulphoxide reductase (MsrB), two structurally unrelated proteins (Kumar *et al*., 2002). A single gene for each of these enzymes is present in the analysed insect species (Table 2).

### Validation by gene expression

The expression of 16 of the 38 antioxidant genes annotated in this paper (*Sod2*, *Sod3*, *Cat*, *Gtpx1*, *Tpx1*, *Tpx3*, *Tpx4*, *GstD1*, *GstS1*, *GstS2*, *GstS3*, *GstO1*, *Trxr-1*, *Trx-like 3*, *Trx/Gtx* and *MsrA*) was validated by their identification in a brain expressed sequence tag library (Whitfield *et al*., 2002). In addition, age and tissue specific expression profiles for eight of these genes (Sod1, Sod2, Cat, Tpx3, Trx-1, GstD1, Gtpx-1 and MsrA) encoding representative members of the main antioxidant families were reported for both workers and queens (Corona *et al*., 2005) (Table 3).

### Bacterial genes

During the annotation of honey bee antioxidant genes, we also found several genes encoding putative bacterial-like antioxidant enzymes, including catalase, Mn SOD, TPX, GST and TRX (Supplementary material, Table 1). In the case of the catalase gene, a fragment was amplified by PCR only in samples from the thorax and abdomen of worker pupae and adult (but not larvae), and was not detectable in worker heads or any body part of adult queens (data not shown). These results suggest that this gene is not integrated into the bee's genomic DNA and might therefore come from endosymbiotic bacteria infecting the digestive tract of the larva. This gene is distinct from the *bona fide Apis* catalase gene discussed above.

We also identified a bacterial-like gene encoding a putative TRX (XP_561198) in the *Anopheles* genomic sequence, which is also presumably the product of bacterial DNA contamination. These examples show that contamination from endosymbiotic bacterial genomes are a common phenomenon present in insect genomic sequence projects, as has been shown for Wolbachia in *Drosophila* species (Salzberg *et al*., 2005).

## Conclusions

We presented the results of manual annotation of the main component of the enzymatic antioxidant system of *Apis mellifera* and a comparative analysis with *Anopheles gambiae* and *Drosophila melanogaster*. This report represents the first systematic comparison of antioxidant genes between insect orders and between social vs. solitary insects. We found that although the basic components of the antioxidant system are conserved, there are important differences in the number of paralogs between species. The main differences include the absence of one of the five members of CuZn SOD family (Sodesque) in bee; duplication of TrxR in fly; expansion of the TRX family in fly; expansion of the Theta, Delta and Omega GST classes in fly and mosquito, and expansion of the Sigma GST class in bee. We have also speculated on how the differential expansion of antioxidant gene families among these species could reflect both differences in their life-style and the quantity of pro-oxidant molecules ingested with the food.

## Experimental procedures

### Annotation of *Apis mellifera* antioxidant genes

#### Identification of putative orthologs

We initially identified genes encoding known components of the enzymatic antioxidant system in organisms with well-characterized genomes, primarily human and *Drosophila melanogaster*. Searches were performed using both key-word searches or protein queries vs. translated DNA databases (tblastn) at NCBI (http://www.ncbi.nlm.nih.gov/), ENSEMBL (http://www.ensembl.org/index.html), and Flybase (http://www.flybase.indiana.edu). Then, we searched the *Apis mellifera* genome for candidate antioxidant genes using the tblastn program with the scaffolds_assembly_2 database at BEEBASE (http://racerx00.tamu.edu/bee_resources.html). This database included a number of gene prediction sets as well as a combined prediction data set (Glean3). Identification of putative antioxidant gene orthologs was completed by multiple protein sequence alignments followed by phylogenetic analysis (see details in next section). As in some cases overall protein homology does not always determine similar function and therefore the identity of an ortholog, additional bioinformatics support for the identification of putative orthologs were performed using the Conserved Domain Architecture Retrieval Tool (CDART) (http://www.ncbi.nlm.nih.gov/Structure/lexington/lexington.cgi?cmd=rps) and by identifying reported conserved residues of the catalytic site for each predicted enzyme.

#### Verification and correction of gene predictions

Verification of automatic gene predictions derived from the honey bee genome project (Honey Bee Genome Sequencing Consortium, 2006) were performed using protein alignments with existing gene prediction sets, selected orthologs (including known isoforms) and if available, EST sequences (http://titan.biotec.uiuc.edu/cgi-bin/ESTWebsite/estima_blastui?seqSet=bee). When conflicts in gene structure were detected between existing gene predictions or with respect to homologs across species, they were resolved using a combination of protein alignments, splice prediction algorithms (http://www.fruitfly.org/seq_tools/splice.html) and manual verification of splicing consensus sequences. A similar approach was followed to build the structure of genes with no automatic predictions (*Sod3*, *Tpx6*).

#### Classification and nomenclature of *Apis mellifera* antioxidant genes

After the identification of a putative *Apis* ortholog, the gene was named following the closest *Drosophila* ortholog. In the case of genes with no assigned names in this *Drosophila* (as in the case of several members of the GST family) we followed the *Anopheles* classification (Ding *et al*., 2003). In the case of bee genes with no identified orthologs in other species, we assigned a name using the family and subfamily abbreviation plus a number (for example, GstS2–4). When members of a gene family have both conserved structural domains and conserved residues of the catalytic site, but are atypical family members (for example, by containing other structural domains) we used in addition the term ‘like’ as in *Trx-like1* and *Trx-like2*.

#### Phylogenetic analysis

Initial protein alignments were performed using CLUSTALW and then edited using the jalview program (http://www.ebi.ac.uk/clustalw/). We removed the predicted N-term and C-term regions when they were extended relative to other homologs in the alignment. Edited sequences were re-aligned using the ClustalX 1.81 program (Thompson *et al*., 1997) with the following parameters. Pair-wise: gap opening = 35.0, gap extension = 0.75; Alignment: gap opening = 15, gap extension = 0.3, protein weight matrix, Gonnet series. Phylogenetic trees were made with the Neighbour Joining method (Saitou & Nei, 1987) using the paup 4.0 b10 program (Swofford, 2002). Trees were rooted using as outgroup the most divergent sequence in each group. The statistical significance of branch order was estimated by the generation of 1000 replications of bootstrap re-sampling of the original aligned amino acid sequences.

### Prediction of subcellular localization

Prediction of subcellular protein localization was performed for all identified antioxidant genes using four programs: PSORT II (http://psort.ims.u-tokyo.ac.jp/form2.html), iPSORT (http://hc.ims.u-tokyo.ac.jp/iPSORT/) (Bannai *et al*., 2002); TargetP (http://www.cbs.dtu.dk/services/TargetP/) (Emanuelsson *et al*., 2000) and SignalP (http://www.cbs.dtu.dk/services/SignalP/) (Bendtsen *et al*., 2004).

## Supplementary material

S1 Deduced protein sequences of bacterial-like antioxidant genes found in the honey bee genomic sequence databases.

This material is available as part of the online article from http://www.blackwell-synergy.com
