# Supplementary material for: Genes of the antioxidant system of the honey bee: annotation and phylogeny
Source: Insect Mol Biol. 2006 Oct 1;15(5):687–701. doi: 10.1111/j.1365-2583.2006.00695.x (PMC1847502; doi:10.1111/j.1365-2583.2006.00695.x)
Supplement: S1 — Deduced protein sequences of bacterial-like antioxidant genes found in the honey bee genomic sequence databases. [file imb0015-0687-ts1.doc]

**Supplementary table 1. -** Deduced protein sequences of bacterial-like antioxidant genes found in the honey bee genomic sequence databases. After the name of each sequence, is indicated the Beebase ID (if assigned) and its localization in the honey bee genomic scaffolds_assembly_2 .
